# Supplementary material for: Intracellular Trafficking and Distribution of Cd and InP Quantum Dots in HeLa and ML-1 Thyroid Cancer Cells
Source: Nanomaterials (Basel). 2022 Apr 29;12(9):1517. doi: 10.3390/nano12091517 (PMC9104504; doi:10.3390/nano12091517)
Supplement: Supplementary file 1 [file nanomaterials-12-01517-s001.zip › nanomaterials-1687316-supplementary.pdf]

Supplementary Data

Dynamic Light Scattering

Dynamic light scattering (DLS) technique was used to characterize the hydrodynamic size of red CdSe/ZnS and InP/ZnS quantum dot dispersion in aqueous solution. QDs samples at 0.1 mg/mL were tested by Colloid Metrix NANO-flex® II with a laser wavelength of 632 nm and scattering angle of 180°.

The DLS results of CZW-R-5 (red CdSe/ZnS QDs) in Supplementary Figure S1 display two peaks at hydrodynamic diameters of 20 nm and 144 nm, while INPW 650-5 (red InP/ZnS QDs) has a monodisperse peak with hydrodynamic diameter around 47 nm (Supplementary Figure S2). The results indicate that there might be the formation of carboxylic acid ligand shells, and the QDs tend to agglomerate when dispersed in aqueous solution.

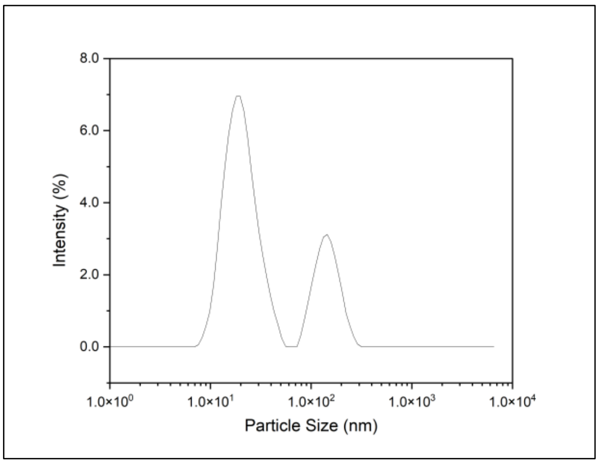

Supplementary Figure S1. Intensity size distribution of CZW-R-5 (red CdSe/ZnS QDs) obtained by Dynamic Light Scattering.

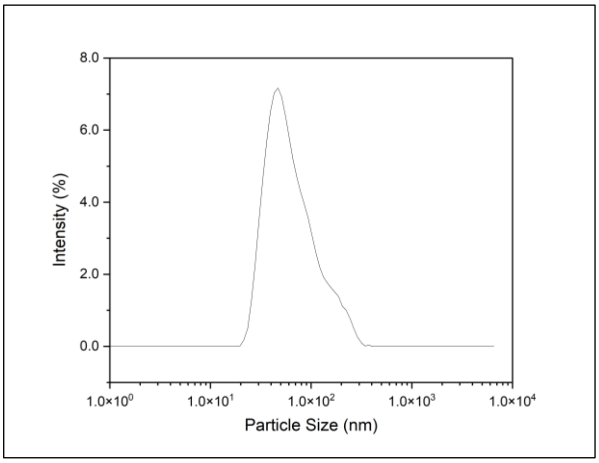

Supplementary Figure S2. Intensity size distribution of INPW 650-5 (red InP/ZnS QDs) obtained by Dynamic Light Scattering.

Scanning Electron Microscopy

A JEOL 7900 field emission scanning electron microscope (FESEM) equipped with a scanning tunneling electron microscopy (STEM) detector was used to image the QDs. Approximately 100 μL of each QD stock solution was diluted to a 1:10 ratio and subsequently sonicated for 15 min to ensure proper separation of QDs in solution. A 5 μL drop of each

QD solution was pipetted onto a holey carbon ultra-thin carbon film copper grid and allowed to air dry for 1 h. The STEM images verify the datasheet values for both CdSe/ZnS (5–10 nm) and InP/ZnS (10–20 nm) (Supplementary Figure S3).

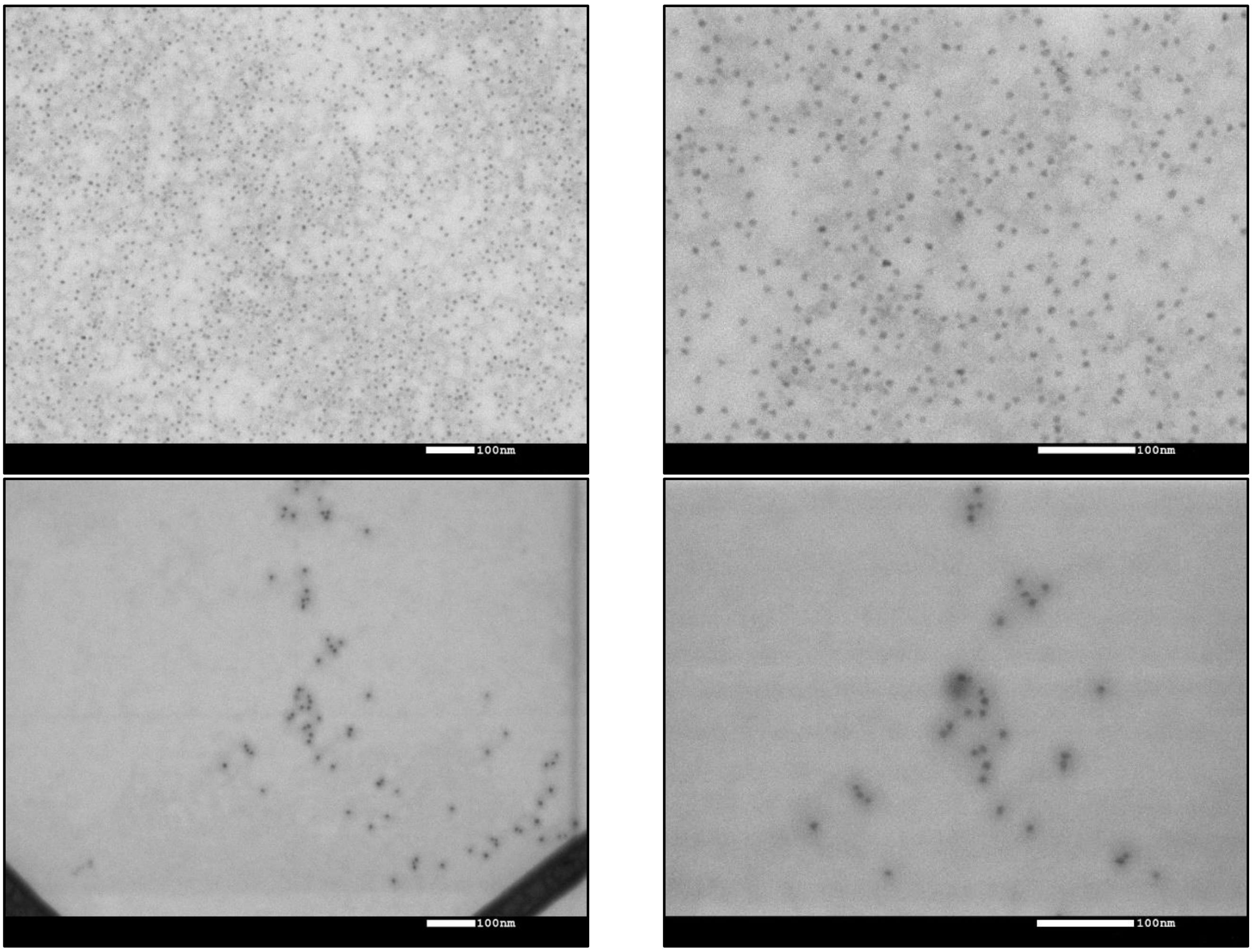

Supplementary Figure S3. Top 2: CdSe/ZnS QDs (Left—100kX, right—200kX) and Bottom 2: InP/ZnS QDs (Left—100kX, right—200kX). Each bar represents a length of 100 nm.

X-Ray Photoelectron Spectroscopy

X-ray photoelectron spectroscopy (XPS) was conducted to verify the elemental and chemical state of each of the two QD materials. Approximately 0.5 mL of each of the stock solutions at 1 mg/mL concentration was pipetted onto a silicon substrate that was placed on a hot plate set to 50 °C. Each QD was pipetted in 50 µL increments, where each aliquot was allowed to dry in between subsequent aliquots to form a film of QDs. A Thermo Scientific Nexsa XPS was used to evaluate the composition of each of the 2 QD materials (CdSe/ZnS and InP/ZnS). XPS data indicated by the peak locations and peak shifts in the binding energy signifies the element and its chemical state, respectively. In the case of the CdSe/ZnS (Supplementary Figure S4/Table S1), the peak positions for the element and the chemical state of each constituent are shown. The same is shown for the InP/ZnS QDs (Supplementary Figure S5/Table S2).

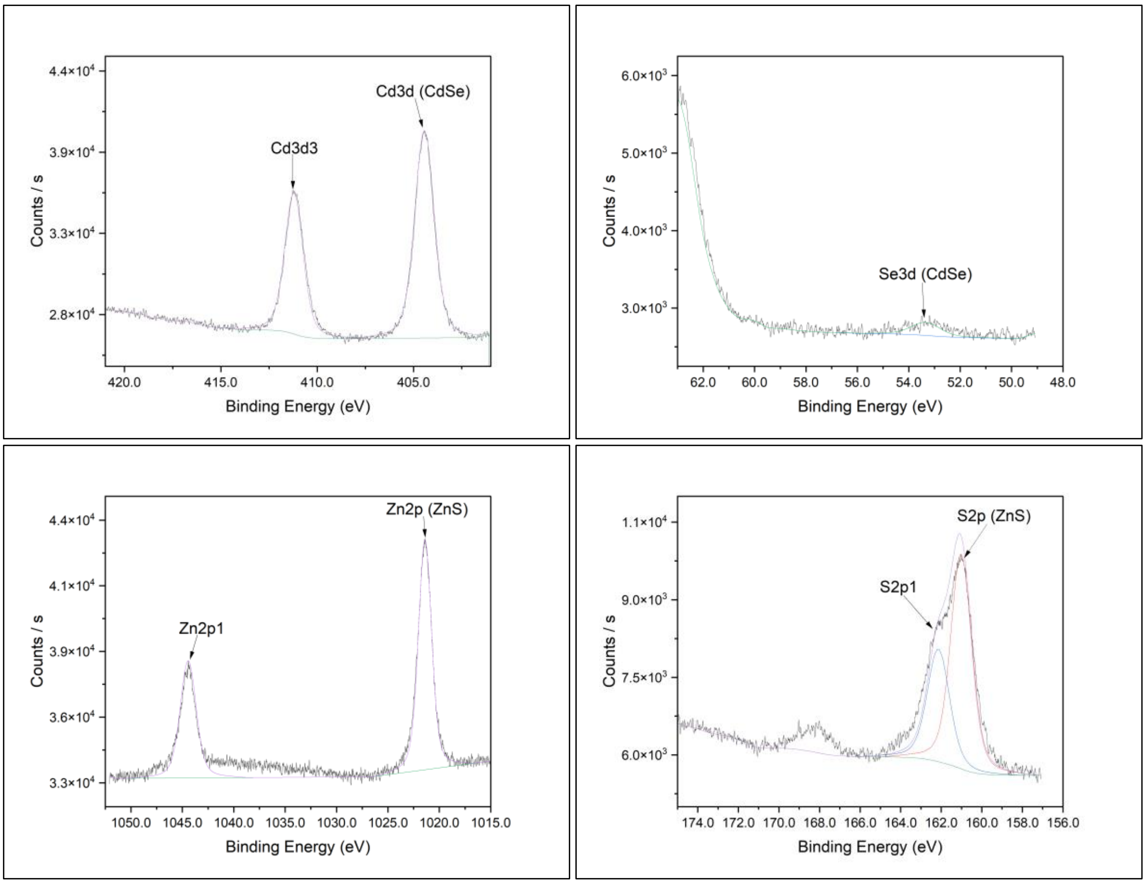

Supplementary Figure S4. XPS scans of CdSe/ZnS QDs of Cd, Se, Zn and S regions.

Supplementary Table S1. XPS peak positions and quantitative composition of CdSe/ZnS QDs.

Name	Peak BE	Height CPS	FWHM eV	Area (P) CPS.eV	Atomic %
Se3d CdSe	53.38	422.64	0.09	597.32	0.93
S2p ZnS	161.24	15472.54	2.23	36544.57	51.65
Cd3d CdSe	404.52	58811.83	1.15	133535.12	14.31
Zn2p ZnS	1021.42	54294.97	1.48	183155.93	33.11

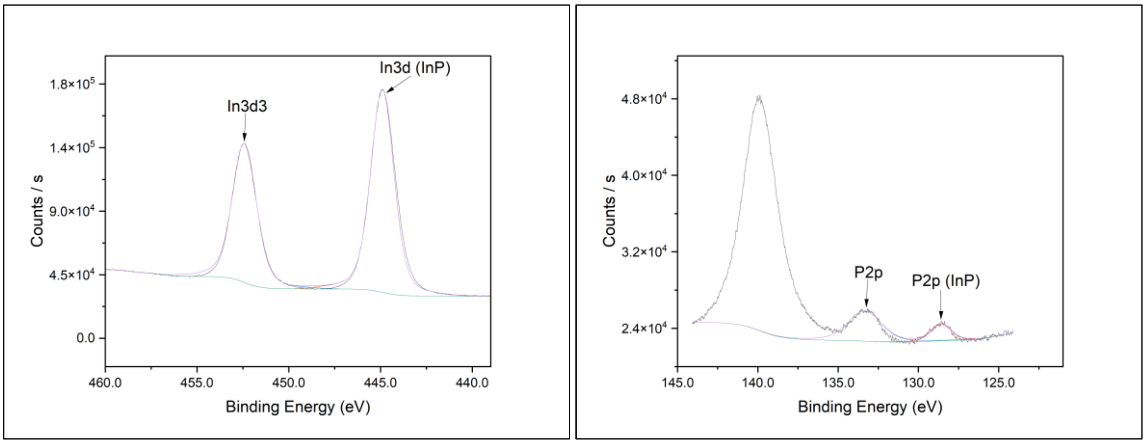

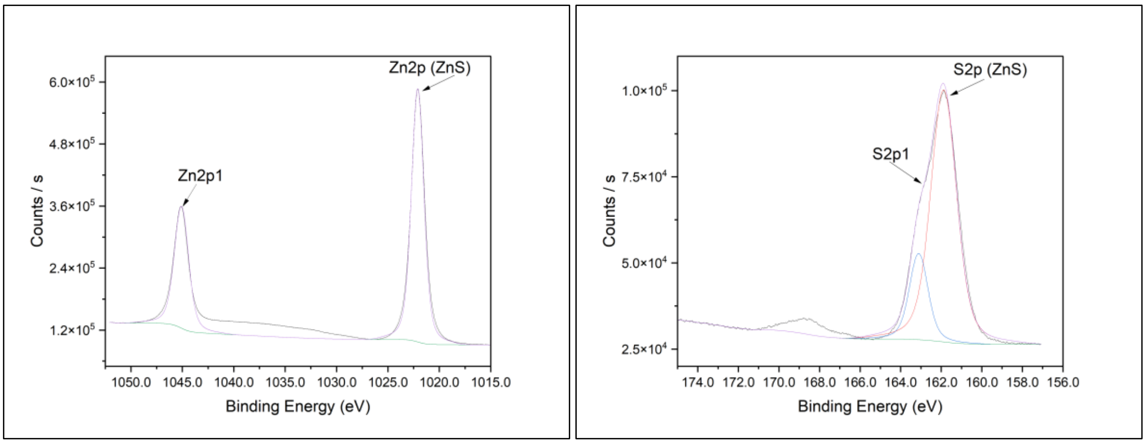

Supplementary Figure S5. XPS scans of InP/ZnS QDs of In, P, Zn and S regions.

Table S2. XPS peak positions and quantitative composition of InP/ZnS QDs.

Name	Peak BE	Height CPS	FWHM eV	Area (P) CPS.eV	Atomic %
P2p InP	133.13	2659.64	1.10	7178.72	2.42
S2p ZnS	161.92	70,730.71	2.14	159,766.96	39.31
In3d InP	444.88	142,421.62	1.56	422,569.51	7.30
Zn2p ZnS	1022.08	486,774.48	1.49	1,618,982.22	50.98

QD Fluorescence via UV Excitation

The stock concentration of QDs was irradiated with UV light source at 365 nm showing subsequent red fluorescence as shown in Supplementary Figure S6.

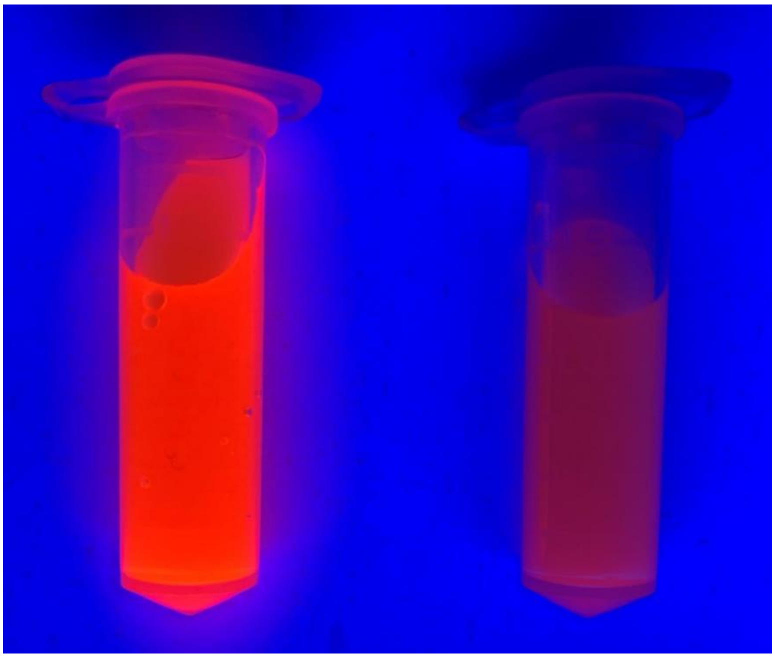

Supplementary Figure S6. UV (365 nm) excitation of CdSe/ZnS (**left**) and InP/ZnS (**right**) QDs.
